# An investigation of physical and mental health consequences among Chinese parents who lost their only child

**DOI:** 10.1186/s12888-018-1621-2

**Published:** 2018-02-12

**Authors:** Qianlan Yin, Zhilei Shang, Na Zhou, Lili Wu, Guangyu Liu, Xiaoqian Yu, Huaihui Zhang, Haidong Xue, Weizhi Liu

**Affiliations:** 1 Faculty of Psychology and Mental Health, Second Military Medical University, 800 Xiangyin Road, Shanghai, 200433 China; 2 Department of psychology, University of South Florida, Tampa, USA; 3 Mental Health Center of Yangpu District, Shanghai, China; 4 Health Family Planning Commission of Yangpu District, Shanghai, China

**Keywords:** Loss-of-only-child family, *Shidu* parents, Mental and physical health, PTSD

## Abstract

**Background:**

The term “loss-of-only-child family” means that the only child in a family passed away or is disabled due to an accident or other events. The parents who cannot conceive or do not adopt another child, are known as *Shidu* parents in China. This study compares the physical and mental health of *Shidu* parents with those parents who have not experienced such loss.

**Methods:**

The target group is comprised of parents being *Shidu* for more than 1 year (*N* = 95) and the control group is comprised of parents with a living child (*N* = 97) from the same area as the *Shidu* parents. Socio-demographic information and physical health outcomes were collected by the adapted questionnaires. PCL-C (PTSD Checklist-Civilian Version), CES-D (Center for Epidemiological Studies Depression Scale) and GHQ-12 (General Health Questionnaire) were applied to evaluate the parents’ physical and mental status.

**Results:**

*Shidu* parents have a higher risk of developing PTSD and depression, and suffer more severe psychiatric disorders compared to parents with a living child. The rate of PTSD in the *Shidu* group was up to 32.6% and the scores of PCL-C are much higher than the control group. The physical status of *Shidu* parents were much worse than that of the control group, characterized by higher morbidity of chronic diseases and more hospital visits.

**Conclusions:**

*Shidu* parents have more severe mental health problems and a higher rate of chronic diseases than parents who have a living child. Loss of the only child is the most traumatic event for the parents, which is a serious and unique problem in Chinese society that deserves attention. More studies and support are desired to improve the physical and mental health of *Shidu* parents.

## Background

“Loss-of-only-child family” means that the only child in a family passed away or is disabled due to an accident, and the mother has passed her reproductive age [1]. These parents are referred to as *Shidu* parents in Chinese society. The number of one-child families in China has exploded in the past 30 years as a result of the only-child policy [2, 3]. To illustrate, among people age 25 to 29 (born in 1980 to 1990), 15% of them are from only-child families. However, the percentage nearly quadrupled among those born after 2000 [4]. According to the population census statistics in 2011, about 76,000 *Shidu* families occur each year [5], and the number of *Shidu* families in China will reach one million in a decade based on the latest demographic statistics. Thus, the challenges this population is facing need to be addressed in order for the government to better assist them.

One obvious challenging this population is facing is the grief caused by losing the only child. Several studies have suggested that sudden death (e.g. from an earthquake or accident) of a loved one is an important risk factor for PTSD [6–10] and depression [10, 11]. Although these studies on parental bereavement have provided significant insights and theoretical perspectives to understand *Shidu* parents, the majority of the studies are about the mental consequences of losing relatives, spouses, siblings and others, very few of them are specifically about losing an only child, which may raise different concerns from losing other family members. This is especially true for Chinese parents. Chinese culture is family-oriented. Children and parents form a very close relationship, and children live with their parents till college or marriage. Parents take care of their young children and children are their aging parents’ main care-giving providers. This is not only a phenomenon stem from cultural awareness but also an obligation enforced by law. For parents, losing the only child often means losing their main source of care-giving, termination of the family line, a great threat to the individual’s well-being [12], and consequences of declining mental health or social withdrawal, which may progress to a point of social isolation because communicating about their child’s death is often difficult and can be hurtful and stigmatizing. According to the studies conducted with the bereaved people, the death of a child results in a significantly high intensity of unresolved grief, which can worsen mental and physical health [13–16]. To date, only one study [17] has been conducted in mainland China to investigate *Shidu*, and it indicates that with reduced family members as a result of the population control, the unexpected death of a beloved one can be more devastating and traumatic for other family members than the days before the population control. Therefore, besides financial support, social support in the form of grief counseling is also needed to prevent the declining of mental health [18, 19]. At the moment, not enough social workers in China can provide such counseling service, and therefore institutes need to be established to train these social workers.

In summary, *Shidu* is a unique social issue in Chinese society after the decades-long one-child policy [20]. Therefore, Chinese government has been aware of the importance of providing support for *Shidu* parents. Considering the growing population in *Shidu, and the* lack of systematic investigation of the health consequences arising in this population*,* our study aimed to examine different facets of health status in *Shidu* parents, thus to add the corresponding assistance from the government.

In the present study, we administered questionnaires to collect data on the physical and psychological health status of *Shidu* parents and parents with a living child in the Yangpu district of Shanghai. We hypothesized that *Shidu* parents would have more severe mental problems and a higher rate of chronic diseases than parents with a living child.

## Method

### Participants and procedure

We obtained information about the *Shidu* parents from the Register of Population Statistics provided by the Health Family Planning Commission of Yangpu District, Shanghai. One hundred and ten out of 800 *Shidu* families were randomly selected from 11 communities of Yangpu district in Shanghai (we only selected one parent from each “loss-of-only-child family” as our participant). We organized a team including a psychologist, a psychiatrist and several psychology graduate students to investigate the *Shidu* parents under help from local social workers knowing the families. A household survey was also administered to the *Shidu* parents, which took approximately 30–40 min to complete. Some participants became emotional during the investigation, we had to pause the investigation to comfort them, thus the entire investigation process can take 1–2 h. The parents were instructed to read the recruitment letter detailing the purpose, method, and procedure of the study, they were assured of anonymity and confidentiality in the reporting of the results, and the right to withdraw from the study anytime. Researcher’s contact information was given out to the participants, research ethics were carefully considered prior to the study. This study was approved by the ethics committees of the Second Military Medical University.

The criteria for the target group are listed below:

1. *Shidu* parents above 49 passed the reproductive age or do not want another baby.
  China’s general birth control policy was transformed into the one-child policy nationwide in 1980’s. As the legal age of marriage is 22 for men and 20 for women, those who complied with the policy would be at the age of more than 49. Therefore in this study, both *Shidu* parents and the parents with a living child were above 49, the latest childbearing age. Besides, *Shidu* parents have not adopted a child by the time they participated in this study.
2. They could read and understand the questionnaires and completed all the questions (parents with serious mental diseases such as schizophrenia were excluded).
3. *Shidu* parents lost their only child 1 year ago.

We performed stratified random sampling according to the ratio of 1:1 in the same area from April to November in 2015. The control group has the same sample size as the target group, and both groups are equally distributed in gender, age and family incomes.

### Measures

Demographic information: gender, age, nationality, place of residence, religious belief, educational background, marital status and subjective assessment of family economic status (according to the average monthly household income in Shanghai in 2015, “below 3000 yuan” is considered as bad; “over 3000 yuan” as good).

PTSD Checklist-Civilian Version (PCL-C) to screen for bereavement-related PTSD, developed by Weathers et al. [21]. We used a self-report instrument that corresponds to the PTSD criteria according to the Diagnostic and Statistical Manual of Mental Disorders, Fourth Edition [22]. Participants responded to 17 items on a 5-point Likert-Type scale (1 = *not at all, and* 5 *= extremely*) in three areas: intrusion, avoidance and numbing, and hyperarousal. The Chinese version of the PCL-C has shown excellent internal consistency and convergent validity with other measures of PTSD severity in previous studies [23].

Depressive symptoms were measured with the 20-item Center for Epidemiological Studies Depression Scale (CES-D) [24]. The CES-D asks subjects how often they experienced a number of situations or feelings in the past week. Possible responses range from 0 (“*rarely or none of the time [less than 1 day]*”) to 3 (“*most or all of the time [5-7 days]*”). The composite scores of the CES-D items range from 0 to 60, with higher scores indicating more frequent symptoms. The internal consistency is 0.84. The measurement has the same validity in China [25] as in Western countries [26].

The General Health Questionnaire (GHQ-12) was used to evaluate the severity of general psychiatric morbidity. As a shortened version of the GHQ-60, it is well adjusted for long-term studies that require an indicator of minor psychiatric morbidity and has proved to be valid in both the clinical and general populations [27]. The Chinese version of the GHQ-12 has sound reliability and validity of the diagnostic performance [28]. The 12 items scored on the basis of 0–0–1-1 (the first 2 answer scores were 0 and the 2 following scores were 1). The total scores range from 0 to 12, with higher scores indicating worse health conditions. The internal consistency of the GHQ-12 in the present study was 0.85.

Questionnaires for physical health outcomes included two items. One is designed for collecting information on whether the participants had common chronic diseases including hypertension, coronary heart disease, diabetes mellitus, cerebrovascular diseases (such as cerebral stroke, congestion and hemorrhage), diseases of digestive system (such as chronic gastritis, enteritis, hepatitis, cholecystitis, gall-stone, and polypus) and respiratory system (such as chronic bronchitis, pneumonectasis, asthma and tuberculosis), rheumatism (such as rheumatic condition and pain in joint), osteoarthropathy (such as hypertrophic, tuberculous and retromorphosis), tumor, mental illnesses and so on. The other item measures the number of outpatient physician visits for physical health or other reasons. It was ranked in 4 levels (1 = *0–2 times*, 2 = *3–5 times*, 3 = *6–9 times*, 4 = *equal to or greater than 10 times*).

### Data analysis

We collected 95 valid questionnaires out of 110 *Shidu* parents according to the criteria (with ten declining our investigation and five failing to meet the criteria). To maintain the same sample size as the target group, 100 families were selected as the control group from the 11 communities, and 97 questionnaires were collected. Analyses were conducted using IBM Statistical Package for Social Sciences version 20. Missing values (less than 5%) were replaced by the sample mean. Chi-square and t tests were carried out for further analysis.

## Results

Demographic information of the two groups is presented in Table 1. Chi-square tests revealed no significant difference between *Shidu* and the control group in terms of gender (*χ*^*2*^ = 0.04, *p =* 0.83), marital status (*χ*^*2*^ = 0.41*, p* = 0.82), educational level (*χ*^*2*^ = 3.26, *p* = 0.20), and religious belief (*χ*^*2*^ = 1.10, *p* = 0.58), but there is a significant difference when it comes to age (*χ*^*2*^ = 8.76, *p* < 0.01) and family income (*χ*^*2*^ = 5.44, *p* = 0.02).

**Table 1:** Demographic Statistics for “Shidu” Group and Normal Group

	Control Group *N* = 97		“*Shidu*” Group *N* = 95		χ^2^	*p*-value
	n	%	n	%		
Gender						
Female	65	67.01	65	68.42	0.04	0.83
Male	32	32.99	30	31.58		
Age						
50–64	47	48.45	66	69.47	8.76	< .001 ***
65–80	50	51.55	29	30.53		
Marital status						
Married	85	87.63	84	88.42	0.41	0.82
Divorced	2	2.06	3	3.16		
Widowed	10	10.31	8	8.42		
Educational Level						
Lower than high school	42	43.30	50	52.63	3.26	0.20
High school	45	46.39	32	33.68		
Over than high school	10	10.31	13	13.68		
Religion Belief						
None	80	82.47	76	80.00	1.10	0.58
Buddhism	11	11.34	15	15.79		
Other	6	6.19	4	4.21		
Family income						
Below 3000	52	53.61	35	36.84	5.44	0.02*
3000 and over	45	46.39	60	63.16		

Note.*p-value< .05; *** *p*-value < .001

Table 2 shows the group differences in PTSD, depressive symptoms, and general health condition. Results from the t-tests indicated that the *Shidu* group scored significantly higher on all the scales than the control group, implying that the *Shidu* parents were more vulnerable to develop PTSD, depressive and mental illness symptoms than the parents with a living child.

**Table 2:** “Shidu” parents Compared With The Normal in the scales

	Control Group *N* = 97		“*Shidu*” Group *N* = 95		t	*p*-value
	Mean	SD	Mean	SD		
PCL-C	23.90	7.29	45.28	15.85	−11.970	< .001***
CES-D	3.47	5.17	16.33	14.64	−8.076	< .001***
GHQ-12	0.58	0.91	3.74	3.90	−7.702	< .001***

*Note. PCL-C* PTSD Checklist-Civilian Version, *CES-D* Center for Epidemiological Studies Depression Scale, *GHQ-12* The General Health Questionnaire

***p-value< .001

On the PCL-C, we set a cut-off of 50 to suggest a PTSD diagnosis [29]. The results showed that the highest score of the *Shidu* was 81, the lowest was 17, and over 50 participants totaled 31. The rate of PTSD in the *Shidu* group was up to 32.6% with 26 females and five males, and the women (PTSD_female_ = 40.0%) had a higher positive rate of PTSD than men (PTSD_male_ = 16.7%). We conclude from these data that the *Shidu* parents had higher morbidity than the control group, and women were more likely to develop PTSD than men.

We collected the morbidity of chronic diseases (which included hypertension, coronary heart disease, diabetes mellitus, cerebrovascular diseases, diseases of the digestive system, diseases of the respiratory system, arthritis, osteoarthrosis, tumors, mental diseases and so on), and the number of visits to a hospital in recent years mainly to assess the level of physical health. We observed a significantly higher overall morbidity of coronary heart disease (*χ*^*2*^ = 23.355,*p* < .01), tumors (*χ*^*2*^ = 4.828, *p* = .028), mental diseases (*χ*^*2*^ = 4.828, *p* = .028) and other unclassified diseases (*χ*^*2*^ = 6.887, *p* = 0.009) in the *Shidu* parents than the parents with a living child from Table 3. According to the total number of visits to the hospital in recent years (see Table 4 for the comparison of the number of visits to a hospital in the two groups), the *Shidu* group had more visits to the hospital than the control group (*χ*^*2*^ = 17.362, *p* = 0.001). 54 (56.8% of the total) *Shidu* parents visited the hospital more than 10 times, while the corresponding number in the control group is 28 (28.9% of the total). In conclusion, *Shidu* parents have worse physical health conditions compared to the parents with a living child.

**Table 3:** The comparison of mortality in the two groups

	Control Group *N* = 97		“*Shidu*” Group *N* = 95		χ^2^	*p*-value
	n	%	n	%		
Hypertension	37	38.1	38	36.5	0.035	0.852
Coronary heart disease	3	3.1	27	28.4	23.355	<.001***
Diabetes mellitus	16	16.5	17	17.9	0.066	0.797
Cerebrovascular diseases	3	3.1	5	5.3	0.153	0.696
Respiratory diseases	4	4.1	6	6.3	0.129	0.720
Digestive diseases	3	3.1	5	5.3	0.153	0.696
Rheumatosis	2	2.1	7	7.4	1.954	0.162
Osteoarthrosis	11	11.3	17	17.9	1.655	0.198
Tumor	1	1.0	7	7.4	4.828	0.028*
Mental diseases	1	1.0	7	7.4	4.828	0.028*
Other	2	2.1	1	11.6	6.887	0.009**

*Note*. *p-value< .05; ** *p*-value < .01; *** *p*-value < .001

**Table 4:** The comparison of the visit time to hospital in the two group

Times	Control Group *n* = 97		“*Shidu*” Group *n* = 95		χ^2^	*p* value
	N	%	N	%		
0–2	33	34.0	18	18.9	17.362	0.001
3–5	28	28.9	14	14.7		
6–9	8	8.2	9	9.5		
> = 10	28	28.9	54	56.8		
Total	97	100.0	95	100.0		

## Discussion

The purpose of the current study was to test the assumption that *Shidu* parents have impaired physical and mental health due to losing an only child by comparing a group of *Shidu* parents to their counterparts with a living child. The comparison was based on a series of standardized assessments of their physical health and mental health problems such as PTSD and depression. The results of this study have confirmed the hypothesis that PTSD and depressive symptoms are more common in *Shidu* parents than the control group.

Children are the major source of joy, hope, meaning, and purpose in life for all parents, and it is the most devastating grief for parents to lose a loved child [30]. The loss of an only child is particularly traumatizing and heartbreaking for Chinese parents as a result of the “one-child” policy and the cultural role that children play in a Chinese family. Although China is becoming more westernized, Confucianism still represent the core values of Chinese society. One of the values is making the passing on the family name an obligation, failing to do so triggers a great deal of stress [31]. Children are the main caregivers of their aging parents [32, 33], and play an indispensable role in maintaining the family line. *Shidu* parents can feel extremely desperate and overwhelmed about the termination of their family line (especially those who could no longer conceive), and they are highly vulnerable to PTSD and depressive symptoms. As a result, systematic and culturally sensitive psychological assistance and mental health services should be provided to this particular group of bereaved parents.

Many studies have recognized the psychological and physical consequences of losing a loved child [14], but very few studies have addressed in detail the physical effects. Our results showed a higher morbidity in coronary heart disease, tumors, mental diseases and other unclassified diseases in the *Shidu* group compared to the control. These diseases were closely related to the long-term psychological suffering of losing the only child. More importantly, the parents in our study became *Shidu* only 1 year ago, so for parents being *Shidu* longer, the physical and mental health consequences could be much more severe than our findings. In summary, it is urgent to offer support to prevent the diseases from worsening in *Shidu* parents. Our findings were consistent with a nationwide follow-up study in Denmark [34] and several studies in China [20] in revealing the difference between the *Shidu* group and control group in terms of health. However, from a clinical view of mental disease, 32.6% of the *Shidu* group in our study have PTSD, much higher than the ratio from relevant studies [35, 36]. Furthermore, our finding that women are more likely to develop PTSD and depression than men echoed western studies, which provide some explanation like the role expectations of women compared to men [35]. Given the role expectation in China, mothers are playing a bigger role in raising up their only child, and spend more time with the child than fathers, so mothers and children have a very closed relationship, therefore losing the only child raises more challenges for mothers than for fathers.

Several limitations in the design of this study should be taken into account when the results were evaluated. First, the actual physical and mental condition can be more severe than the results reported here, as some *Shidu* parents rejected the investigation since it was emotionally challenging for them to talk about the bereavement. Second, the sample were recruited from one area, which might have limited the ability to generalize to people from other areas with a different social-economic status. Although we have already included a larger scale than the previous studies, the sample should be extended considering the large population of *Shidu* parents in China. Third, there were differences in demographic information in the two groups. Poor health condition and syptoms of PTSD were more likely to be observed among the *Shidu* group even though the *Shidu* group were wealthier and younger than their counterparts. However, the differences didn’t impact the results in a significant way, instead those indirectly supported our attribution of sever experience of losing the only child mainly damaging their physical and mental health. In fact, although the data collection process was not as smooth as expected due to the sensitive and emotional topic of Shidu, we were still able to collect ample samples with the help of the committee workers.

To summarize, this study has provided a picture of the current health condition of *Shidu* parents, and we hope to stimulate the development of corresponding regulations and services to improve their life quality. For instance, the findings have demonstrated the need to enhance health care to *Shidu* parents, and to intervene with those who did not go to clinical services regularly or were unwilling to get clinical services. Further investigation is needed to explore some other predictors responsible for the prevalence of PTSD and depression in *Shidu* parents, such as the personality of the parents, the characteristics of the lost children, and the social environment [37]. More studies can be beneficial to address the factors causing the differences between the *Shidu* parents and the parents with a living child.

## Conclusion

*Shidu* parents have more severe mental health problems and a higher rate of chronic diseases than their counterparts who have a living child. Losing the only child is the most traumatic event for senior parents who cannot conceive, which is a serious and unique problem in Chinese society due to the one-child policy. More support is needed to help *Shidu* parents to improve their health status, and more studies are desired to address the problems and challenges this population is facing, in order to better their life quality.

## Abbreviations

CES-D: Center for Epidemiological Studies Depression Scale
GHQ-12: General Health Questionnaire
PCL-C: PTSD Checklist-Civilian Version
PTSD: Post-trauma syndrome disease
